# Child and environmental risk factors predicting readiness for learning in children at high risk of dyslexia

**DOI:** 10.1017/S0954579416000134

**Published:** 2017-02

**Authors:** Julia Dilnot, Lorna Hamilton, Barbara Maughan, Margaret J. Snowling

**Affiliations:** a University of Oxford; b St. John's College; c York St. John University; d King's College London

## Abstract

We investigate the role of distal, proximal, and child risk factors as predictors of reading readiness and attention and behavior in children at risk of dyslexia. The parents of a longitudinal sample of 251 preschool children, including children at family risk of dyslexia and children with preschool language difficulties, provided measures of socioeconomic status, home literacy environment, family stresses, and child health via interviews and questionnaires. Assessments of children's reading-related skills, behavior, and attention were used to define their readiness for learning at school entry. Children at family risk of dyslexia and children with preschool language difficulties experienced more environmental adversities and health risks than controls. The risks associated with family risk of dyslexia and with language status were additive. Both home literacy environment and child health predicted reading readiness while home literacy environment and family stresses predicted attention and behavior. Family risk of dyslexia did not predict readiness to learn once other risks were controlled and so seems likely to be best conceptualized as representing gene–environment correlations. Pooling across risks defined a cumulative risk index, which was a significant predictor of reading readiness and, together with nonverbal ability, accounted for 31% of the variance between children.

It has been known for many years that dyslexia runs in families, and there is accumulating evidence of its association with candidate genes (Paracchini, Scerri, & Monaco, 2007). Thus, the prevalence of dyslexia is elevated in the offspring of parents with reading difficulties (e.g., Pennington & Lefly, 2001; Scarborough, 1990; Snowling, Gallagher, & Frith, 2003). However, the interpretation of these familial effects is not straightforward because of the interplay of genes and environment in contributing to reading outcomes (van Bergen, van der Leij, & de Jong, 2014).

When considering the role of genetic and environmental factors in determining literacy outcomes, particularly important are gene–environment correlations (the influence of parental genes *working through* the environment; Plomin, DeFries, & Loehlin, 1977; Scarr & McCartney, 1983). Because a parent's genotype correlates with both the child's genotype (here a genetic risk of dyslexia) and the environment provided by the parent for the child (say, a poor literacy environment), an example of a passive gene–environment correlation (passive *r*GE), it is not surprising that parental reading accounts for a small but significant amount of variance in the reading outcomes of children at family risk of dyslexia, over and above a child's own cognitive skills (e.g., Carroll, Mundy, & Cunningham, 2014). There are two further types of *r*GE that should be considered. First, an evocative *r*GE correlation in which children who have inherited a genetic risk for dyslexia may evoke less literacy-related input from their parents than those without a family risk and an active *rGE* correlation in which children who have a heritable propensity for dyslexia select environments in which there is little exposure to print.

A second, and possibly related, risk factor for dyslexia is a preschool language impairment (for a review, see Bishop & Snowling, 2004); many children at family risk of dyslexia experience delays and difficulties with speech and language development (e.g., Scarborough, 1990), and many “late talkers” have parents who report a history of reading difficulties (e.g., Duff, Reen, Plunkett, & Nation, 2015). Here we investigate the noncognitive risks associated with being at high risk of dyslexia either because of a family history of reading problems or because of a preschool language impairment and the predictors of “readiness to learn.” We are particularly interested in whether family risk of dyslexia accounts for variance in children's reading readiness and attention and behavior at school entry, once other important contextual and environmental factors are controlled.

There are a small number of reports of subtle differences between the home literacy environments experienced by children at family risk of dyslexia compared with those not at risk: van Bergen, de Jong, Maassen, and van der Leij (2014) found less shared reading between fathers with dyslexia and their children compared with controls, and Torppa et al. (2007) found less frequent book, newspaper, and magazine reading by parents in at-risk families (arguably a passive *r*GE) and more variable measures of shared reading when the children were 2 years of age (a possible active *r*GE). In addition, Scarborough, Dobrich, and Hager (1991) reported that parents of children who went on to be dyslexic attributed limited shared storybook reading to their children's lack of interest in books (which could be construed as an evocative *r*GE).

It is well established that there is a social gradient in reading attainment, and socioeconomic status and parental education level are predictors of literacy outcomes (for a review, see Phillips & Lonigan, 2005). More specifically, the home literacy environment has been found to be associated with early reading and may at least in part mediate the effects of socioeconomic status (Sénéchal & LeFevre, 2002).

To our knowledge, there is only one study that goes beyond home literacy environment to examine whether other contextual and home factors predict outcomes for children at family risk of dyslexia. In this study, Aro et al. (2009) measured parental influences defined by a composite including mother's education level, father's unemployment, parental sensitivity at 14 months, support for joint attention, self-reported affection in parenting, general stress (described as “risks” in their paper), and parenting-related stress and depression symptoms when children were aged 4. They proceeded to investigate the impact of these influences in addition to family-risk of dyslexia status and neurocognitive risks on a range of outcomes at 8–9 years. Children in the family-risk group were subject to more risks in the “parental” and “neurocognitive” risk domains than the children not at family risk of dyslexia. For IQ, neurocognitive risk but neither group status (family risk vs. control) nor parental risk was a predictor. There was a different pattern for reading fluency, which was predicted by family-risk status and neurocognitive risk but not by parental influences. Finally, parental risk domain but not family-risk status predicted social adaptation, and neurocognitive risks accounted for a small amount of further variance.

Following Aro et al. (2009), we investigated the possible association of child, environment, and family factors with familial risk of dyslexia and how these factors predict child outcomes around the time of school entry using data from a longitudinal study of children at high risk of dyslexia and controls. In terms of outcomes, we defined school readiness by two measures: (a) reading readiness, which is a composite of early word reading, phoneme awareness, letter–sound knowledge, and rapid automatized naming at 5.5 years; and (b) behavior and attention at 4.5 years, which is parental ratings of children's externalizing behaviors. Together, these outcomes comprise a set of skills and behaviors that children are expected to have in place to benefit from schooling; we call these “readiness for learning.”

Our study differed from that of Aro et al. (2009) in several ways. First, the families came from a wider range of socioeconomic circumstances and included not only children at family risk of dyslexia determined by parental status but also children whose parents were concerned about their preschool language development. Second, we focused on the point of school entry, an earlier stage of development, before a downward spiral can magnify differences in literacy and other scholastic skills between children who are identified as dyslexic and those who do not have reading problems. Third, instead of using one parental risk variable, which included a broad range of parenting risks, we assessed multiple indices in order to ascertain which family and child risks are important for predicting readiness to learn. Although this design confounds genetic risks with environmental influences passing between parents and children in biological families, we can make some progress in understanding the combined influence of genes and environment using this approach.

We drew on existing literature guided by the bioecological framework of Bronfenbenner and Ceci (1994) to identify a wide range of risk factors that have been found to be associated with poor school attainment. These included, as a distal influence, socioeconomic status (Phillips & Lonigan, 2005); as proximal influences, the home literacy environment (Bradley & Caldwell, 1976; Koury & Votruba-Drzal, 2013; Melhuish et al., 2008) and family stresses (e.g., parental mental health; Cogill, Caplan, Alexandra, Robson, & Kumar, 1986; Grace, Evindar, & Stewart, 2003); and at the individual level, child health risks (e.g., premature birth; Chen, Claessens, & Msall, 2014) and gender, with boys typically having poorer reading and being more susceptible to reading difficulties than girls (Rutter et al., 2004). Finally, children do not experience risks in isolation, and one way of capturing the overall risk status of a child is to sum the number of risks to which they are exposed (Evans, Li, & Whipple, 2013; Luthar, 1993). Generally, the greater the cumulative risk, the more negative the developmental outcomes for the child, as illustrated by research on outcomes including IQ (Sameroff, Seifer, Barocas, Zax, & Greenspan, 1987), school achievement in adolescence (Gutman, Sameroff, & Eccles, 2002) and externalizing behavior problems (Appleyard, Egeland, van Dulmen, & Sroufe, 2005; Deater-Deckard, Dodge, Bates, & Pettit, 1998; Greenberg, Speltz, DeKlyen, & Jones, 2001), and in children at family risk of dyslexia, in cognitive, academic, and social adaptive outcomes (Aro et al., 2009). In this light, we expected that an index of cumulative risks would account for variance in “readiness to learn” once general cognitive abilities were controlled.

In summary, we sought to investigate the effects of factors at different contextual levels, namely, distal (socioeconomic status), proximal (home environment and family stresses), and child (health risks), on the development of readiness to learn at the end of the preschool period. Further, we asked which of these variables makes a unique contribution to outcomes when all other risks are taken into account and, specifically, whether family risk of dyslexia will show independent links with readiness to learn when proximal and distal factors are controlled.

We used data from a longitudinal study of children at high risk of dyslexia and controls to test the following hypotheses:

*Hypothesis 1:* (a) Family risk of dyslexia and (b) preschool language impairment will be associated with a wide range of environmental and child-level risk factors. *Hypothesis 2:* Risks will co-occur and correlate with the readiness to learn outcomes; an index of cumulative risk will correlate more strongly with outcomes than any single risk factor.
*Hypothesis 3:* (a) Socioeconomic status and home literacy environment will predict reading readiness at school entry; (b) socioeconomic status and family stresses will predict behavior and attention at school entry; and (c) male gender and poorer child health will have a negative effect on developmental outcomes.
*Hypothesis 4:* A measure of cumulative risk will account for variance in reading readiness when general cognitive ability is controlled.

## Method

### Participants

Data are reported from the first three phases of the XXX Project that traced the language and literacy development of children at family risk of dyslexia, children with preschool language difficulties, and controls. The main aim of the project was to investigate the nature and overlap between dyslexia and specific language impairment. The study assessed a wide range of child, parental, and environmental variables at approximately annual intervals from preschool through the early years. Ethical clearance for the study was provided by the University of York, Psychology Department Ethics Committee, and the NHS Research Ethics Committee. Parents provided informed written consent for their child to be involved.

Families were recruited via advertisements placed in local newspapers, nurseries, and the webpages of support agencies for children with reading and language difficulties and via speech and language therapy services. Sample size was determined by a power calculation based on prior family-risk studies. Large effect sizes were expected for the comparison of outcomes between children at family risk and controls (*d*s = 1.18–1.37 for literacy) and between children with speech difficulties and controls (*d* = 0.93). The sample size was determined to provide 90% power to detect a difference of 0.54 *SD* between the risk and control groups (α = 0.05 two tailed).

The sample represented a broad range of socioeconomic backgrounds (mean age at which parents left full-time education was 19 years). None of the children recruited to the sample met exclusionary criteria (monozyotic twinning, chronic illness, deafness, English as a second language, care provision by Local Authority, and known neurological disorder such as cerebral palsy, epilepsy, and autism spectrum disorder). Following recruitment, each parent who consented, regardless of whether or not he or she self-reported as dyslexic, was assessed to ascertain family-risk status (see below). In nine cases, family risk of dyslexia was based solely on the fact that an older sibling had the clinical diagnosis. Children were then classified according to whether or not they met research criteria for specific language impairment (for further details, see Nash, Hulme, Gooch, & Snowling, 2013). Sixteen children referred because of language concerns and who did not meet inclusionary criteria are included in the current sample in the control group.

The children were assessed at six time points: Time 1 (T1; age 3.5), Time 2 (T2; age 4.5), Time 3 (T3; age 5.5), Time 4 (age 6.5), Time 5 (age 8), and Time 6 (age 9). At T1, 245 children were recruited: an additional 15 entered the sample at T2, creating a total sample of 260. Data are analyzed from T1 (3.5 years), T2 (4.5 years), and T3 (5.5 years). Of the 260 children, there were nine sibling pairs. One sibling from each pair was excluded at random, leaving 251 children (149 males, 102 females) in the sample reported here: family risk (FR; *N* = 90); language impairment (LI; *N* = 36); FR+LI (*N* = 37); control (*N* = 88). There was a small amount of attrition between time points (*N* = 18).

### Measures

At T1 and T2 data were collected at the participants' homes using multiple collection methods including parent questionnaires, interviews, and child and parent assessments. Assessment sessions took approximately 2 hr with appropriate breaks, and normally two home visits were required. At T3 data were usually collected in the school setting, and parents completed postal questionnaires.

#### General cognitive ability

Children's general cognitive ability (performance IQ) was estimated from performance on two tests from the Wechsler Preschool and Primary Scale of Intelligence (Wechsler, 2003): block design (α = 0.85) and object assembly (α = 0.90) given at T1. Composite nonverbal IQ scores were calculated based on the mean of *z*-standardized scores for the two subtests.

#### Risk Indices

##### Family risk of dyslexia

The procedure used for determining family-risk status was based on previous studies. These have primarily used parental self-report measures. However, we considered it appropriate to validate this procedure with objective assessment when possible because it is not uncommon for parents with a history of reading difficulties to be unaware that they have dyslexia. Thus, children were classified as at family risk if (a) a parent self-reported as dyslexic on the Adult Reading Questionnaire (Snowling, Dawes, Nash, & Hulme, 2012); (b) a parent scored below 90 on a literacy composite of nonword reading and spelling; (c) a parent had a discrepancy between nonverbal ability and the literacy composite of 1.5 *SD*, with a literacy composite standard score of 96 or below; or (d) a sibling had a diagnosis of dyslexia from an educational psychologist or a specialist teacher. In the current sample, for 96 families family risk status was based on one affected family member (44 mothers, 43 fathers, and 9 siblings); in the remaining 31 families, it was based on two or more first-degree affected members.

This is a dichotomous risk index with 1 = *family risk of dyslexia*, 0 = *not at family risk of dyslexia*, so negative correlations between family risk of dyslexia and readiness for learning are expected.

##### Socioeconomic status.^1^

The index of socioeconomic status included father's education, mother's education, father's occupation, and mother's occupation. Each of the variables was standardized and the standardized *z* scores summed and divided by four to produce an index of socioeconomic status. For education, the measure was number of years in education after the age of 14; for occupation, the level was coded according to the Office for National Statistics (2010) categories. The highest ever occupational level rather than the current occupational level at T1 was used in order to capture information about parents who were currently full-time caregivers. The index was coded so that a higher score represents a greater socioeconomic status risk, that is, lower socioeconomic status.

##### Home literacy environment

The index of home literacy environment at T2 included storybook exposure, frequency of story reading, number of children's books in the home, and adult author checklist (a measure of primary caregiver's book exposure). The four measures were standardized, summed, and divided by four to create a measure of the home literacy environment. The resulting *z* score was multiplied by –1 so that a higher score indicates higher risk. Storybook exposure score consists of scores on two checklists in which targets had to be discriminated from foils: the Children's Title Checklist and the Children's Author Checklist (Hamilton, 2013). The Children's Title Checklist consisted of 30 titles of popular children's books and 30 plausible foils. The Children's Author Checklist consisted of 40 popular children's book authors and 40 foils. For both checklists, primary caregivers were asked to “check the box next to every title/author that they recognized.” Frequency of storybook reading was the sum of the parent's responses to two separate items: “How many times do you, or other members of your family, read stories to or with your child at bedtime in a typical week?” and “How many times do you, or other members of your family, read stories to or with your child at other times during the day in a typical week?” Number of children's books in the home was estimated by parents on an ordinal scale ranging from 0–20 to >200. The Adult Author Checklist consisted of 40 authors of contemporary fiction, representing a broad range of genres, and 40 foils.

##### Family stresses

The family stresses index included scores at T1 and T2 for the primary caregiver's health and psychological well-being on the General Health Questionnaire (Goldberg & Williams, 2000) and reports of stressful life events experienced by the child. Stressful life events recorded included bereavement, parental separation, or serious illness (moving house and the birth of a sibling were not included). The measures were standardized, summed, and the total divided by four to create a mean *z* score for family stresses.

##### Child health

The child health index included premature birth, birth complications, hearing problems, visual problems, physical difficulties, current health problems, early health problems, and significant accidents. The score for each variable (0 = *no*, 1 = *yes*) was summed to create an index with a theoretical maximum value of 8. This variable was then standardized. The questionnaire items (T1) were *premature birth*, “Was your child born before 37 weeks?”; *birth complications* “Were there any unusual complications at birth?”; *hearing problems* “Is your child's hearing within normal limits?”; *visual problems* “Is your child's vision within normal limits?”; *significant accidents* “Has your child had any serious injuries or accidents, for example, head injuries or broken bones?”; *current physical difficulties* “Have there ever been concerns about your child's physical development?”; *early health difficulties* “Were there any unusual complications in early childhood?”; and *current health difficulties* “Is your child's health good at present?”

#### Cumulative risk index

To estimate the number of risks experienced by each child, the following steps were taken. First, each of the continuous variables included in the socioeconomic status, home literacy environment, and family stresses risk indices was transformed by placing a cut at the 15th percentile of the distribution for the sample, excluding those at family risk, in order to create a dichotomous variable: risk/no risk (the child health risk variables were already dichotomous). Second, a categorical risk index was derived for each of the socioeconomic status, home literacy environment, and family stresses indices by summing the number of risks within each index. To achieve equal weighting to the other categorical risk indices, the eight risk variables describing child health were weighted 0.5 (rather than 1) when they were summed. Thus, for each categorical risk index, the maximum possible value was 4. Finally, to create the cumulative risk index, the categorical risk indices were summed for each child to give a hypothetical maximum of 16 risks.

#### Outcome measures

##### Reading readiness

Four measures were used to derive a reading readiness (T3) outcome measure: early word reading (α = 0.98), letter–sound knowledge (α = 0.95) and phoneme deletion (α = 0.91; York Assessment of Reading for Comprehension; Hulme et al., 2009), and a rapid automatized naming task (Denckla & Rudel, 1976) in which the children were asked to name pictures of objects as quickly and accurately as possible (test–retest = 0.71). Raw scores were standardized, summed, and divided by four to create a mean *z* score.

##### Behavior and attention

The measure of behavioral and attention was a composite of the hyperactivity (intraclass correlation = 0.42) and conduct (intraclass correlation = .23) subscales of the Strengths and Difficulties Questionnaire (Goodman, 1997), completed by parents at T2. The total score was standardized and multiplied by –1 so that larger scores represented better performance.

## Results

Although the study recruited children with language difficulties into one group, language skills were also reasonably well distributed in the sample. Scrutiny of the data revealed that the distribution of the risk indices conformed to normality except for child health, which was positively skewed. We used nonparametric correlations for examining the relationships between this and the other measures.

Our analysis plan was designed to test the main hypotheses. To test Hypothesis 1 we examined whether risks were associated (a) with family risk of dyslexia and/or (b) preschool language impairment. Using data from the whole sample, we tested Hypothesis 2 by assessing the relationships between the individual risk factors at different levels (distal, proximal, and child) and a measure of cumulative risk for males and for females. We next proceeded to assess the contribution of the individual risks to outcomes using regression analysis to test Hypothesis 3, and to investigate whether family risk accounted for unique variance in readiness to learn once other risks had been controlled. A final model assessed Hypothesis 4 to ascertain whether a measure of cumulative risk would account for variance in reading readiness or attention and behavior when general cognitive ability was controlled.

### Group differences in risk indices

Preliminary analyses found that the numbers of risks experienced by boys and girls did not differ significantly. Table 1 shows descriptive statistics for the risks and outcomes according to FR and LI status, pooled across gender and family statistics for univariate analyses.

**Table 1.** Risk indices, general cognitive ability, and behavioral outcomes according the family-risk and language status

	No Family Risk				Family Risk					
	TD Control		LI		FR		FR+LI		*F* (1, 160)	
	Mean	*SD*	Mean	*SD*	Mean	*SD*	Mean	*SD*	FR	LI
Risk indices^*a*^										
Socioeconomic status^*a*^	−0.53	0.52	0.15	0.82	−0.01	0.66	0.39	0.64	9.67**	19.47***
Home literacy environment^*a*^	−0.38	0.53	0.21	0.77	−0.11	0.67	0.44	0.48	4.61*	23.86***
Family stresses^*a*^	−0.28	0.91	0.10	0.91	0.03	0.99	0.13	0.77	<1	<1
Child health^*a*^	−0.22	0.81	0.30	1.21	−0.08	0.94	0.23	0.94	<1	5.57*
Cumulative risk^*b*^	1.6	1.48	3.04	2.25	4.44	3.44	4.00	2.41		
Outcomes^*a*^										
Reading readiness (T3)^*a*^	0.39	0.72	−0.41	0.89	0.02	0.67	−0.60	0.73	5.95*	37.34***
Attention & behavior (T2)^*a*^	0.13	0.78	−0.04	0.68	0.03	0.86	−0.46	0.97	3.78†	4.29*
General cognitive ability										
Performance IQ at T1	114.20	14.04	96.36	13.35	108.01	14.77	100.08	11.55		

*Note:* TD, Typically developing; LI, language impairment; FR, family risk; T1–T3, Times 1–3.

^*a*^The values are *z* scores.

^*b*^Max = 16.

†*p* = .05. *p < .05. **p < .01. ***p < .001.

In the upper rows of the table, it can be seen that the control group (first column) has low rates of risk and the children at high risk of dyslexia (FR, LI, and FR+LI) are exposed to relatively more risks. Generally, the pattern is for the FR-only group to experience fewer risks than the LI-only group and the FR+LI group to be subject to the most risks. The only exception to this pattern was for child health risks; these were more common in the LI-only than the FR+LI groups. A multivariate analysis of variance found that there was a significant effect of FR, *F* (4, 157) = 2.53, *p* < .05, and of LI status, *F* (4, 157) = 8.64, *p* < .001, on risk indices and the interaction between FR and LI was not significant, *F* (4, 157) < 1. A series of univariate analyses of variance (far right columns) showed that children at FR of dyslexia were of lower socioeconomic status and had poorer home literacy environments than children not at risk, but there were no group differences in family stresses or child health risk. LI status had a significant effect on socioeconomic status, home literacy environment, and child health risk indices but not on the index of family stresses.

The lower rows of Table 1 show the data relating to outcomes. Again there is a “step” pattern, with the typically developing group having better outcomes than the “risk” groups and among the risk groups the FR-only doing better than the LI and then the FR+LI group. A 2 × 2 between groups analysis revealed that the effect of family-risk status was significant on reading readiness and marginally nonsignificant on attention and behavior (*p* = .05); the effect of LI status on both reading readiness and attention and behavior was significant. The interaction between FR and LI was not significant for either outcome measure (*p*s > .24), indicating that these risks were additive. Given that the influences of family risk of dyslexia and language impairment on outcomes were independent, we went on to investigate how distal, proximal, and child-level risks are associated with readiness for learning outcomes, alongside family risk of dyslexia.

### Relationships among risk factors and developmental outcomes

Although the above analyses treat risk indices as independent, risks do not occur in isolation. Table 2 shows the relationships between the risk indices and cumulative risk (above the diagonal for boys and below the diagonal for girls, respectively). It can be clearly seen that the risk indices are positively intercorrelated. In particular, there are strong correlations between socioeconomic status and home literacy environment risk indices (*r*s = .45–.64) while the correlations between the child health risk index and the other indices and between home literacy environment and family stresses indices are low. In most cases, the *r* values are larger for boys, but none of the differences between male and female correlations were statistically significant, and so gender was not controlled in the further analyses.

**Table 2.** Correlations between continuous risk indices and PIQ (above the diagonal for males and below for females)

	1	2	3	4^*a*^	5	6
1. Socioeconomic status		.638	.286	−.019	.764	−.339
2. HLE	.445		.123	.035	.653	−.315
3. Family stresses	.126	.169		.107	.589	−.315
4. Child health^*a*^	.197	.043	.293		.366	−.048
5. Cumulative risk	.650	.515	.615	.562		−.471
6. PIQ	−.057	−.131	−.066	−.246	−.150	

*Note:* PIQ, Performance IQ; HLE, home literacy environment.

^*a*^These correlations are nonparametric; all other correlations are parametric.

Table 3 shows the correlations between the risk indices and the outcome measures. Generally, the higher the child's scores on the risk indices (i.e., the higher the risk), the poorer “readiness for learning.” Correlations between the child health index and each of the outcome measures are low. Reading readiness is correlated moderately with socioeconomic status and home literacy environment risk indices and with the cumulative risk index, whereas the correlations with family stresses are low. Similarly, attention and behavior shows a moderate degree of correlation with socioeconomic status, home literacy environment, and cumulative risk and correlates with the family stresses index. As predicted, the index of cumulative risk shows a higher correlation with each of the outcome measures than any of the other risks in isolation.

**Table 3.** Correlations among continuous risk indices, PIQ, and readiness for learning outcomes

Risk Index	Reading Readiness	Attention and Behavior
Socioeconomic status risk	−.261	−.232
Home literacy environment risk	−.291	−.268
Family stresses risk	−.138	−.236
Child health risk^*a*^	−.122	−.163
Cumulative risk	−.365	−.313
PIQ	.434	.086

*Note:* PIQ, Performance IQ.

^*a*^These correlations are nonparametric; all other correlations are parametric.

### Predictors of readiness to learn

Because different risks co-occur, it is important to investigate which of these predict “readiness for learning” when the other risks are taken into account. A parallel set of hierarchical regression analyses with missing data excluded pairwise investigated this issue, entering risk indices in successive blocks according to theoretical assumptions regarding the proximity of the different risks to the outcomes. Socioeconomic status (the most distal factor) was always entered into the first step, followed in the second step by home literacy environment and family stresses (proximal factors), and in the third step, child health (a child-level factor). To investigate whether family risk of dyslexia accounted for independent variance in readiness for learning once the influences of the environmental and child health variables were taken into account, family risk of dyslexia was entered as a dummy variable in the last step, together with interactions between family risk and the other risk indices. There was no evidence for any interaction between family-risk status and any of the other risk indices; therefore, these interactions were not included in the final models.

Table 4 shows the findings of these analyses for the two outcome measures. The β values are presented for each factor in each step of the hierarchical regression. These values change as more risks are added to the model because of the covariance between risks. In Model A, socioeconomic status was a significant predictor of both readiness to learn outcomes when entered into the first step of the model. At the second step, home literacy environment was also a significant predictor of both outcomes; for attention and behavior, family stresses accounted for additional variance. Of note, socioeconomic status was no longer a significant predictor of either outcome when home literacy environment and family stresses were included in the model. At the third step, child health was significant as a predictor of reading readiness but not of attention and behavior. Family-risk status was not a significant predictor of either outcome in the final step of this full multivariate model. We ran a further set of analyses in which we dropped the nonsignificant predictors from the initial models and then entered family-risk status at a second step (see Table 4, Model B). In the model predicting reading readiness, home literacy environment and child health accounted for 11% of the variance and family risk for a further 2.8%, which was not significant. In the model predicting attention and behavior, home literacy environment and family stresses accounted for 11% of the variance at the first step and family risk accounted for no further variance. These analyses confirm that family-risk status is not a significant predictor of either measure of “readiness to learn” when other distal, proximal, and child risk factors are taken into account.

**Table 4.** Hierarchical regression models predicting readiness for learning

			Reading Readiness		Attention and Behavior	
	Step	Factor	β	*p*	β	*p*
Model A^*a*^	1	Socioeconomic status	−0.261	.001	−0.232	.003
	2	Socioeconomic status	−0.125	.171	−0.082	.363
		Home literacy environment	−0.209	.021	−0.195	.030
		Family stresses	−0.083	.272	−0.192	.012
	3	Socioeconomic status	−0.118	.192	−0.076	.398
		Home literacy environment	−0.210	.019	−0.195	.029
		Family stresses	−0.053	.490	−0.164	.033
		Child health	−0.152	.045	−0.137	.068
Model B^*b*^		Home literacy environment	−0.236	.000	−0.237	.001
		Family stresses			−0.202	.005
		Child health	−0.162	.012		
		Family risk of dyslexia	−0.118	.072	−0.014	.840

^*a*^Total *R*^2^ = .127 for reading readiness and = .135 for attention and behavior.

^*b*^Total *R*^2^ = .118 for reading readiness and = .103 for attention and behavior.

Finally, we assessed whether the noncognitive risks that we evaluated here continued to account for “readiness to learn” over and above known predictors of educational attainments. We ran two parallel analyses, one predicting reading readiness and one predicting attention and behavior, entering the cumulative risk index together with a measure of general cognitive ability (nonverbal IQ at T1) as predictors. Nonverbal IQ significantly predicted reading readiness (*R*^2^ = .18) but not attention and behavior; the cumulative risk index predicted both outcomes (reading readiness *R*^2^ = .03; attention and behavior *R*^2^ = .07). Together these two factors assessed in preschool predicted 31% of the variance in reading readiness and 10% of variance in attention and behavior at school entry.

## Discussion

In this study, we evaluated the role of distal (environmental), proximal (family), and child risk factors in predicting readiness for learning at school entry in children at high risk of reading difficulties. The high-risk sample comprised children with a family history of dyslexia and children with preschool language difficulties (approximately half of whom also were at family risk of dyslexia) and typically developing children. A key question was whether family risk of dyslexia explains variation in reading readiness and attention and behavior when more distal risks are controlled.

In line with Hypothesis 1, we found that children at family risk of dyslexia as children experienced more risks likely to affect their development than typically developing controls, consistent with Aro et al. (2009), and this also applied to children with preschool language difficulties. The risks included factors known to affect reading attainment, namely, lower socioeconomic circumstances and a less rich home literacy environment, those associated with family stresses and health problems affecting the child. Moreover family risk of dyslexia and preschool language impairment were additive risk factors such that children who were both at family risk of dyslexia and language impaired accumulated more environmental and health risks.

As predicted by Hypothesis 2, we found that risks tended to co-occur. There were strong correlations between socioeconomic status and home literacy environment; correlations were lower between family stresses and child health and between these and the other variables. There were no gender differences in risks, and the correlations between risks were not significantly different for males and females. Similarly, there were no gender differences in outcomes. As expected, there was a negative relationship between risks and outcomes, such that the more risks a child experienced, the poorer was their “readiness for learning” in school. Further, in line with our hypothesis, an index of cumulative risk correlates more strongly with outcomes than any single risk factor, though the differences in correlation were not statistically significant.

Examining individual risks further, we confirmed that socioeconomic status and home literacy environment are predictors of reading readiness (Phillips & Lonigan, 2005; Hypothesis 3a). However, when entered together in the model, the effect of socioeconomic status falls from significance, suggesting its effects on reading readiness are mediated by home literacy environment. Over and above the effects of home literacy environment, child health, but not family stresses, is a significant predictor of reading readiness, and together they account for 11% of the variance in reading readiness. Once these risk indices are controlled, being at family risk of dyslexia contributes no further variance to outcomes. Similarly, both socioeconomic status and family stresses predict our measure of behavior and attention (in line with Hypothesis 3b), and socioeconomic status is not a significant predictor when home literacy environment is in the model. Together, home literacy environment and family stresses accounted for 11% of the variance in attention and behavior, and being at family risk of dyslexia explains no further variance in outcome.

It follows from these findings that family risk of dyslexia should not be taken to imply genetically mediated effects per se. While previous studies have suggested that family-risk status is a predictor of literacy outcomes (e.g., Puolakanaho et al., 2007; Snowling et al., 2003; Torppa et al., 2007; van Bergen, de Jong, Plaka, Maassen, & van der Leij, 2012), in most cases the majority of variance is accounted for by child-cognitive variables. For example, Carroll et al. (2014) reported that after controlling for earlier reading and language skills, family risk of dyslexia accounted for 3.1% of variance in reading accuracy. Using a large sample of families not selected for dyslexia risk, van Bergen, Bishop, van Zuijen, and de Jong (2015) reported that parental reading fluency accounted for 5% of the variance in children's reading skills after controlling for children's own phonological awareness, rapid naming, and visual attention span. In this light, our finding that family-risk status did not account for variance in outcomes when contextual factors, including those likely to be expressed via *r*GEs, suggests that the residual variance in previous studies might be environmental in origin. Moreover, the lack of interactions between family-risk status and the other risk indices replicates the finding of Aro et al. (2009) that children at family risk of dyslexia are not differentially affected by the number of risks. However, this does not rule out the possibility that such interactions could be demonstrated in a genetically sensitive design.

The influence of home literacy environment on school readiness (and emergent reading in particular) is well established (e.g., Bradley & Caldwell, 1976; Hamilton, 2013; Koury & Votruba-Drzal, 2013; Melhuish et al., 2008). The current findings replicate those of family-risk studies showing that home literacy environment (including parental reading skills) explains variance in literacy outcomes over and above a child's own cognitive skills (Torppa, Lyytinen, Erskine, Eklund, & Lyytinen, 2010). The reason for its impact on attention and behavior is less clear, though it is possible that interactions with print during the preschool years provide a calm opportunity in which a child develops the ability to self-regulate. The home literacy environment, however, is unlikely to be purely a “passive” influence that the child receives; it may also reflect active and evocative *r*GEs. For example, if a child is well behaved and enjoys listening, then a responsive parent is likely to read more often with that child than would otherwise be the case. The current sample included children with preschool language difficulties; it would not be surprising if such children evoked fewer interactions involving language and literacy than those with typical language.

A novel finding was that a measure of early and concurrent child health accounted for a small but significant amount of variance in reading readiness. When each of the child health risks is entered into the model in the place of the overall index, only one indicator accounts for unique variance in reading readiness: hearing problems at T1 (5.5% of variance). Furthermore, although reports of hearing concerns were more frequent in all of the high-risk groups, it was children at family risk of dyslexia who *also* had preschool language difficulties who were most susceptible to these. Although we do not have objective data and our findings should be treated with caution, further research into the possible causal association between such risks and reading attainment is warranted.

In line with our hypothesis, family stresses and home literacy environment are predictors of attention and behavior at the end of the preschool period. In keeping with this, it is well known that maternal depression and family stress is associated with externalizing behavior problems (e.g., Appleyard et al., 2005; Deater-Deckard et al., 1998; Grace et al., 2003). Moreover, if family stresses are low, then there will be more time for quiet reading and reciprocal effects on children's self-regulation. The absence of an association between behavior problems and family-risk status per se is consistent with the findings of Bonifacci, Montuschi, Lami, and Snowling (2014), who showed that there was no difference in stress levels between the families of children with dyslexia and controls (see Carroll, Maughan, Goodman, & Meltzer, 2005).

Despite the undoubted importance of extrinsic factors on children's propensity to learn, the models including all of the risks explained only a modest amount of variance in what we describe as “readiness to learn.” It is reasonable to assume that cognitive variables explain much of the missing variance. With this in mind, we investigated how much variation in school readiness outcomes could be accounted for by a combination of general cognitive ability (a marker of cognitive risk) and cumulative risk (a marker of noncognitive risk). Performance IQ and cumulative risk are both significant and unique predictors of reading readiness as we predicted, and together they account for 31% of its variation. IQ, in contrast, is not a predictor of attention and behavior.

Our findings extend those of Aro et al. (2009) to an English sample with a broader range of socioeconomic circumstances and to an earlier developmental stage. There are several reasons why children at family risk of dyslexia may experience more risks than children not at risk in the preschool years. These include the possibility that lower socioeconomic status is a downstream effect of poor parental educational attainments of parents with dyslexia, and commensurate with this, they tend to have poorer career opportunities and less well-paid forms of employment (Maughan, 1995). There are also likely to be active, passive, and evocative *r*GEs contributing to the associations found. For example, parents with dyslexia spend less time reading for pleasure than adults who are not dyslexic, and children carrying a genetic risk of dyslexia may evoke less literacy-related (and hence oral language) input from their caregivers than those who do not carry a familial risk. The current design does not allow us to differentiate active/evocative from passive effects, so we do not know their relative influence. More generally, it is unclear whether parental literacy should itself be considered an index of genetic risk rather than a measure of environmental variability. This is an important topic for future research.

Our sample overrepresented children at high risk of dyslexia and underrepresented those with psychosocial adversities. Moreover, because the cutoffs for the cumulative risk measure are sample dependent, it difficult to generalize the findings with regard to this index to the population at large; it also needs to be acknowledged that the way in which family risk is defined will influence findings. Despite these limitations, the current study serves to remind us that being at family risk of dyslexia does not just imply that a child comes to the task of reading with a genetic propensity to find reading difficult. Rather, being at family risk of dyslexia appears to confer a wider range of environmental risks than much previous research on children at familial risk of dyslexia has assumed. Differences in both reading readiness and attention and behavior already present at school entry set the stage for failure not only to learn to read but more generally across the curriculum.

### Conclusions

Children at family risk of dyslexia are exposed to more risks than children not at family risk, but family risk alone is less strongly associated with readiness for learning than other contextual and child-health factors and does not account for any variance in outcomes once risks associated with these other factors have been taken into account. Family risk of dyslexia is thus best conceptualized, not purely as a proxy for genetic risk, but as reflecting gene–environment interplay. The home literacy environment is an important predictor of reading readiness, together with child health, and it also predicts attention and behavior together with family stresses. The significance of these findings for public health points to the importance of the early years in offering children the best chances in education. Moreover, they suggest potential for interventions that support parents in providing a rich home literacy environment to help their children with emergent reading skills.

## Footnotes

1. The measures within the socioeconomic status, child health risk, and family risk of dyslexia indices were based on accepted criteria for these risks. The remaining eight variables that were hypothesized to increase the likelihood of poor school readiness outcomes were entered into a principal component analysis with direct oblimin rotation. Two components (corresponding to home literacy environment and family stresses) had eigenvalues over a Kaiser criteria of 1 and in combination explained 45% of the variance.
